# Understanding the synergies among factors shaping parents' intention to use walking school buses

**DOI:** 10.1038/s44333-026-00126-z

**Published:** 2026-09-02

**Authors:** Khatun E. Zannat, Maximiliano Lizana, Judith Y. T. Wang, David P. Watling

**Affiliations:** 1 Institute for Transport Studies (ITS), University of Leeds, Leeds, West Yorkshire UK; 2 School of Civil Engineering, Universidad de La Frontera, Temuco, Chile; 3 Department of Civil Engineering, University of Leeds, Leeds, West Yorkshire UK

**Keywords:** Environmental social sciences, Psychology, Psychology

## Abstract

Walking School Bus (WSB) programmes offer a promising approach to promoting active school travel (AST). However, there is limited consensus on how shifts from conventional travel modes to WSB will unfold. Existing AST research has examined determinants of walking and cycling, but relatively little attention has been given to WSB and similar emerging modes. This study addresses this gap by developing the Intended Mode Choice Adoption (IMCA) framework to examine the combined effects of behavioural, contextual, and socio-demographic factors on parental intentions. Survey data from 192 parents in Bradford, UK, were analysed. Results indicate that perceived behavioural control and subjective norms toward WSB, attitudes toward active travel, and satisfaction with existing travel arrangements are key determinants of parental intention to switch to WSB. These factors are influenced by distance, escorting patterns, household, and parental attributes. The findings highlight the importance of integrating behavioural constructs across both conventional and emerging modes.

## Introduction

Walking School Bus (WSB) is a promising programme to promote active school travel (AST). In a WSB, a group of children walk to school under adult supervision along a predetermined route, where they are picked up and dropped off at the set stops in accordance with a schedule. The implementation of WSB has been initiated and sustained in many countries, including New Zealand, Australia, Canada, the United States and several European countries, including Spain, Denmark, and the United Kingdom^1–8^. Evaluations of ongoing and experimental trials of WSB highlighted numerous benefits for the participating children, such as physical, mental, and social well-being, as well as the development of practical life skills and improved understanding of road safety^9–11^. Reduced car use, lower emissions near schools, and increased pedestrian safety are further benefits^3^. Despite its advantages, the widespread adoption of WSB programmes remains limited and has often been confined to pilot initiatives. For example, in the United States, only 6.2% of primary schools implemented a WSB by 2009–2010, up from 4.2% in 2008–2009^12^. More recent evidence from a large evaluation of 184 active WSB programmes across the U.S. and internationally during 2017–2018 shows that WSB initiatives continue to operate, particularly in low-income communities where programmes tend to have more route leaders and stronger integration with other active travel activities^2^. In the Auckland region of New Zealand, 42% of schools have included a WSB in their school travel plans as part of a regional government initiative^3^, although approximately one-third of these planned programmes have yet to be implemented. To facilitate the mainstream integration of WSB as an AST alternative, several critical milestones must be achieved: (1) the development of a demand-responsive WSB framework that prioritises children’s safety, security, and comfort to enable large-scale implementation, (2) the incorporation of WSB options into parents’ mode choice decisions, particularly within the context of their daily activity-travel patterns, and (3) the establishment of sustainable WSB operations that are adaptable to local conditions, including weather and community-specific factors^1,9,13,14^.

In recent years, the literature on AST has made notable strides in examining the potential benefits of WSB programmes^10,11,15–17^, the barriers to their implementation and sustainability^1,2^, and critical design considerations^14,18,19^. Despite the clear positive attitudes towards WSB, there is no consensus on how the shift from conventional school travel modes to WSB will unfold, nor on the factors influencing parents’ mode choice decisions when WSB is offered alongside alternatives such as private cars, walking, and public transport. Although existing research on AST has identified a range of contextual factors (e.g., availability of travel options and environmental conditions)^20–24^, individual-level constraints (e.g., car ownership)^25,26^, and psychological determinants (e.g., attitudes, evaluations, and motivations)^27–30^ that influence parents’ intentions to choose conventional active modes like walking and cycling, these studies have largely overlooked the specific case of WSB^31^, despite the fact that the concept of WSB is different from individual children walking to school. This gap raises critical questions about the predictability of demand for such innovative programmes and challenges foundational assumptions and the validity of current implementation strategies. For example, in conventional school travel demand models, walking mode choice is typically driven by travel time, distance from home to school, infrastructure quality, and parental safety perceptions. In contrast, WSB participation depends on additional factors such as proximity to WSB stops, schedule, volunteer availability, and institutional support. Consequently, conventional demand models may omit critical determinants of WSB participation, reducing their predictive accuracy. Importantly, no study to date has investigated the causal pathways between variables associated with the emerging modes and other available school travel modes prior to developing a predictive model to understand demand for such innovative solutions. However, it is important to acknowledge that the success of promoting WSB schemes depends not only on the effectiveness of the service itself, but also on how parents weigh it against other conventional travel alternatives and the extent to which those alternatives meet their school-travel needs^13^. In other words, variables affecting parents’ intentions to adopt an emerging form of school travel are shaped by their perceptions of the benefits and convenience of both existing and emerging alternatives, which may influence their decision to use or avoid modes such as the WSB. In the AST literature, traditional behavioural models such as the Theory of Planned Behaviour (TPB) have been applied or extended to understand parents’ school travel mode choice in which parents have full knowledge and experience of different school-travel alternatives. However, these models are limited in their ability to incorporate parents’ attitudes, perceptions, and other psychological factors related to an emerging mode, while simultaneously accounting for the dynamics of existing travel choices.

To address the research gap, the present study contributes to extending existing state-of-the-art behavioural theories and the AST literature by exploring parental intentions to adopt an emerging mode, i.e., WSB. While previous quantitative studies have examined factors affecting AST behaviours more generally, this is the first study—to our knowledge—to quantitatively assess parents’ preferences regarding participation in a WSB as part of their existing daily activity-travel patterns. To achieve this, we propose a conceptual framework for intended mode choice adoption (IMCA) of an emerging mode like WSB, which extends the state-of-the-art TPB by incorporating relevant assumptions from the Model of Goal-Directed Behaviour (MGB) and the theory of cognitive dissonance (TCD). Given that WSB interventions offer a promising AST strategy to support children’s well-being and that the success of WSB programmes depends heavily on uptake by individual families, it is essential to examine how behavioural constructs relate to parents’ intentions to adopt this new mode. Understanding these relationships is critical to enabling sustained behavioural change among children and identifying effective targets for future interventions.

Therefore, the contribution of this article is threefold:

- It proposes the IMCA model, which enables the examination of behavioural intentions for services that are novel or not yet widely available. This framework integrates behavioural constructs related not only to the emerging mode but also to other available alternatives.
- It demonstrates the application of the IMCA model using empirical evidence from Bradford, UK. In many UK urban settings, home-school distances are typically short, making walking—whether independently, with a parent, or as part of a WSB—a feasible option for a substantial proportion of families. Applying the IMCA model in the Bradford context enables the development of a framework for promoting WSB schemes that may be transferable to other similar settings, where the vast majority of primary school children either walk or are driven to school and where motorised school-bus provision is largely limited to rural areas or pupils living beyond statutory walking distances.
- It identifies key factors that future WSB interventions should target to increase WSB uptake.

## Literature review

Given that contemporary AST literature indicates that parents’ school travel decisions are shaped by a combination of psychological determinants and external factors, influencing both their current behaviours and their willingness to adopt emerging mobility options, a semi-structured literature review strategy was undertaken to examine these dimensions in depth. Searches were performed in major academic databases including Scopus, Web of Science, and Google Scholar. The search strategy combined keywords related to (i) AST and WSB, (ii) influencing factors, and (iii) conceptual models of travel behaviour. Core search terms included in (i) were “walking school bus”, “active school travel”, “active commuting to school”, “school travel mode choice”. Regarding (ii), we used words such as “determinants”, “factors”, “built environment”, “parental perceptions” and “psychosocial factors”. Finally, for (iii), we focused on “behavioural models” and related terms. Boolean operators (AND/OR) were used to refine the search (e.g., “active school travel” AND “determinants”; “walking school bus” OR “active school travel”). The review included peer-reviewed journal articles published in English, primarily from 2000 onwards, focusing on children’s travel behaviour, interventions promoting active transport, and associated environmental, social, and psychological determinants. The selection process followed an iterative approach, combining keyword searches with backward and forward snowballing (screening reference lists and citing articles) to identify additional relevant studies. A brief overview of parents’ mode choice intentions is provided alongside relevant conceptual models, with a summary of key factors presented in Table 1. In interpreting this body of literature, however, it is important to acknowledge that the existing evidence on AST and WSB programmes is geographically uneven, with a strong concentration of studies conducted in the Global North, particularly in North America, Europe, and Australia.

**Table 1:** Factors influencing parents’ adoption of AST for their children

Broad theme	Research focus	Factors	References
Psychological	Association between psychological factors and other observed and unobserved attributes affecting school travel decision-making	Self-efficacy, attitude, subjective norms and values, desire, satisfaction, intention	^27–30,40–48,51,52,54,55,58,59,67,108^
Individual and family characteristics	Differences in rates of AST among heterogeneous socio-demographic groups	Individual characteristics: Children - age, gender, BMI Parents - marital status, employment, education Family characteristics: Ethnicity, household size, number of siblings, home ownership, income, car ownership, family member’s schedule constraint	^25,26,29,65,66,76,109^
Environmental characteristics	The extent to which environmental attributes—both perceived and objectively measured—facilitate or restrict AST	Neighbourhood and school characteristics - land use mix, road density, junction density, route directness, dead-end density, accessibility, safety and convenience Home-to-school route characteristics - street facilities, major roads, and walkability Other natural features: Weather, slope, elevation	^20,22,24,33,79,110,111^
Activity-travel characteristics and habits	Sensitivity of AST to contextual and structural change	Travel time, cost, distance, repeated behaviour	^21,32,39,40,44,77,79,112^

### Psychological factors affecting parents' mode choice intention

Parents’ school travel mode choice decision-making is a complex process. Earlier studies on parental mode choice primarily focused on the sensitivity to mode-specific attributes such as travel time/cost^21,32,33^, environmental factors^22,34–36^, and socio-demographic characteristics^37^. More recent models emphasise psychological factors that help explain why adequate infrastructure alone does not translate into higher AST participation^38,39^. Within behavioural theories, these factors are viewed as key determinants of intention and subsequent behaviour^40^.

AST studies—across systematic reviews and qualitative and quantitative studies—repeatedly identify individual-level psychological constructs as central influences on school travel behaviour. One of the strongest predictors identified is the perceived ability to perform AST. Self-efficacy, which refers to an individual’s belief in their own capability to perform a behaviour, and perceived behavioural control, which reflects the perceived ease or difficulty of performing that behaviour, are commonly used to denote parents’/children’s confidence in engaging in AST. With few exceptions, most studies report a positive association between perceived ability and engagement in AST, a relationship observed among both parents and children^10,40–44^.

Beyond ability-related constructs, attitude toward AST—defined as parents’ or children’s positive or negative beliefs and evaluations of walking or cycling to school—has also been examined extensively. Similar to ability, several studies have found a positive association between favourable attitudes toward AST and actual participation^45–48^, whereas others find no significant relationship^44,49,50^. These inconsistencies often depend on socio-demographic factors, travel mode examined, and the specific attitudinal dimension considered^51^. For example, Leslie et al.^52^ and Corral-Abós et al. ^46^ found that enjoyment of walking or cycling to school was positively associated with AST only among boys, while Stark et al.^45^ showed that attitudes predicted walking but not cycling. Panter et al.^48^ further demonstrated that attitudinal influences on cycling vary with commuting distance.

Psychological constructs also include subjective norms, defined as parents’ perceptions of social approval or expectations from significant others—such as partners, extended family, peers, teachers, or other parents—which shape their intentions and behaviours. This construct reflects the extent to which parents believe that important people in their social environment support or expect AST^53^. Several studies have reported a positive association between strong pro-AST subjective norms and a greater likelihood of choosing AST, suggesting that perceived social endorsement can meaningfully shape school travel behaviour^40,54,55^.

While prior AST studies have examined the importance of social ties, no studies have explored the potential for positive mass effects—where increased use of a travel mode enhances its attractiveness to future users. These effects influence behaviour not through direct changes in perceived utility, but through psychological and social factors shaping behavioural intention^56^. Such mass effects occur when observing others using a mode signals that it is safe, common, or socially endorsed, thereby increasing its perceived legitimacy and encouraging additional families to adopt it. Similarly, information shared within social networks helps overcome limited knowledge about lesser-used modes. As more people use a mode, its visibility and social connections expand, increasing the likelihood that non-users are exposed to and influenced by users of that mode. Recent mode choice literature also highlights the importance of satisfaction with travel and its association with chosen travel mode^57^. However, very few studies on AST incorporate this factor, despite evidence showing positive relationships between parents’ and children’s AST choices and their satisfaction, either in terms of repeating the behaviour or overall life satisfaction^58,59^. Moreover, the role of desire—defined as a state of mind in which an individual has a personal motivation to perform an action—as a mediator between attitude and intention has been acknowledged in the mode choice literature^60^. However, it has been rarely applied in the AST literature to understand parents’ mode choice decisions.

Studies on AST highlight that while parents acknowledge the value of using active modes for their children’s school commute, actual evidence of a mode shift remains limited^61,62^. Several interventions have been implemented to promote AST, but their effectiveness varies. In the broader context of car use, it is generally agreed that although infrastructural and legislative policy measures can incentivise reduced car usage, psychological strategies targeting attitudes and perceptions are typically more acceptable and cost-effective^63^. Research also suggests that interventions relying solely on improvements to the built environment may not sufficiently change travel behaviour^51^. Designing successful interventions to influence travel behaviour requires a thorough understanding of the decision-making processes of travellers. This involves considering not only environmental conditions but also travellers’ attitudes, preferences, beliefs, intentions, values, and norms^64^. Therefore, the effectiveness of campaigns or interventions depends on identifying and targeting potentially modifiable psychological constructs underlying AST.

### Other factors affecting parents' mode choice intention

In the field of AST literature, several studies have examined parental intentions and decision-making processes in selecting school travel modes^27,32,61,62,65^. These investigations have been approached from various disciplinary perspectives—including transportation, urban planning^23,24^, public health^66^, and behavioural science^27,29,67,68^. Despite the diversity of approaches, the findings consistently underscore the association of multiple factors influencing AST^69^.

Some studies sought to identify factors affecting AST under broad thematic categories. For example, Davison et al.^70^ identified key determinants spanning individual (child and parent), family, school, social, and physical environmental characteristics. Ahlport et al.^71^ classified the factors into three broad categories: intrapersonal and interpersonal characteristics of parents and children, environmental features of the neighbourhood, and school-based environmental and policy factors. Similarly, Stewart et al.^72^, using both quantitative and qualitative methods, identified eight influential factors: distance to school, parental fear of strangers and danger, family schedule constraints, culture, resources, values, neighbourhood and school characteristics, and weather. Other studies have focused more narrowly on specific determinants and explored their sub-categories in detail. For instance, Pont et al.^73^ categorised environmental factors into physical, economic, sociocultural, and political dimensions and analysed their association with broader active travel behaviour among children. Moreover, several studies have extended behavioural theories to include habits of both parents and children as well as other psychological, socio-demographic and facilitating conditions, to explain children’s AST behaviour.^39,40,44^.

In addition to identifying and grouping relevant factors, some studies have assessed their significance, relative importance, and interdependencies^21,40,74–78^. These investigations sought to understand which factors are more influential in specific contexts or how certain factors interact synergistically with others. For example, a study by Tang et al.^79^ identified the importance and ranking of factors affecting AST in a mountainous city case, ranging from school commute distance to household income. Similarly, Mehdizadeh et al.’s^80^ cross-sectional study explored the contrasting effects of determinants such as wealth, gender, and school type between the Iranian and Western European contexts. Such studies are crucial for designing targeted interventions that account for contextual heterogeneity. Understanding these determinants and their combined effects on travel behaviour is essential for effective AST promotion.

### Factors affecting parents' intention to adopt emerging school travel mode

Much of the existing literature on factors influencing parents’ mode choice decisions focuses on conventional transport modes. These include non-motorised modes (walking and cycling) and motorised modes (car, public transport, school bus)^21^. Few studies have investigated the factors that influence parental intentions or choices regarding emerging school-travel modes such as the WSB or bike bus, even though these alternatives are increasingly recognised for their potential to promote AST^13^. Other motorised emerging modes may include ride-hailing services for children or organised car-pooling schemes. However, equating the determinants of emerging modes with those of conventional modes—for example, comparing walking with the WSB, cycling with a bike bus, or ride-hailing and car-pooling with private car use for school travel—would be misleading for several reasons. In this study, the distinction between the WSB and walking has been highlighted, and the subsequent sections address WSB in detail. Comparable analyses could also be applied to other emerging travel modes and their conventional counterparts.

First, WSB is a supervised walking scheme, which eliminates the need for individual parental supervision and addresses safety concerns related to traffic and strangers that typically arise when children walk or cycle independently^12^. Second, WSB operates on a fixed schedule, with designated stops and routes, similar to a school bus. In contrast, walking with adult supervision is usually more flexible, with fewer scheduling constraints beyond arriving at school on time^14^. Third, depending on the implementation model, WSBs can operate across multiple routes, allowing parents to drop their children at a nearby stop^3^. This enables children who live farther from school to participate. Finally, WSB routes may be intentionally longer than direct walking or cycling routes in order to include more children^10^. These routes are often designed to avoid high-traffic or high-emission areas and to offer educational or engaging walking experiences. Adopting a WSB therefore requires both parents and children to understand how the service operates, assess its compatibility with existing family routines, and evaluate their capacity to adapt to it. It also depends on the availability of supporting infrastructure, safety measures, and organisational resources.

### Conceptual models for intended mode choice adoption

In the field of AST literature, the study of the relationships between psychological constructs, behavioural intention and observed behaviour has been traditionally conducted following conceptual models grounded in well-established psychological theories. This approach has been successful in recognising the complex set of relationships among psychological constructs such as individuals’ attitudes, subjective norms, beliefs, satisfaction and desire that significantly influence mode choices^27^. Theories such as the TPB^81^, MGB^82^ and TCD^83^ have been applied to different extents in the literature. Among them, the TPB has been the most widely and successfully applied theory to understand mode choice behaviour through the analysis of the role of attitudes, a trend also observed in studies investigating the school travel behaviours of both parents and children^29,69^.

According to the TPB, the intention to perform a behaviour is the most immediate predictor of that behaviour. Intention itself is shaped by three components: attitude, subjective norm, and perceived behavioural control, each of which stems from an underlying belief structure. For instance, behavioural beliefs about the outcomes of an action influence attitudes toward that behaviour. Nonetheless, it has been argued that the TPB’s reliance solely on these three predictors may not sufficiently capture the complexity and variability of actual behaviour. As a result, some researchers have extended TPB by integrating additional psychological variables to construct more comprehensive theoretical models^40,67^. One such extension is the MGB by Perugini and Bagozzi^82^, which introduces satisfaction by measuring positive and negative feelings at the same level as the three predictors stated in the TPB. At the same time, desire acts as a mediator of the effect between the four constructs and intention. These additions have been validated, though on a limited scale in mode choice studies. For example, De Vos et al.^60^ found that attitudes toward public transport positively influence the desire to use public transport, and that this desire, in turn, positively affects intended public transport use. Despite these findings, the influence of desire has yet to be incorporated into AST studies aiming to understand school travel behaviour.

Furthermore, both TPB and the MGB primarily consider unidirectional relationships between variables. Such unidirectional paths may be sufficient when all variables relate to the same choice of interest or are complementary, e.g., attitudes and desires toward active travel. However, the complexity increases when examining how variables may conflict or change. For instance, how might a positive attitude toward active travel and active travel intention be influenced by a desire for car ownership within a household? Or, how might satisfaction or negative feelings change the intention and, in return, change attitudes? This suggests the potential for bidirectional or reciprocal relationships, as proposed by the TCD^83^, which posits that satisfaction can influence and be influenced by attitude.

While these theories provide valuable foundations for understanding both school-travel and other travel-behaviour contexts, their constructs often require adaptation and integration to develop conceptual models that better capture the complexity of real-world decision-making. Such a contribution would help overcome the complexities specifically generated when the behavioural intention in question is related to a service that is novel or not readily available. It would also provide a more comprehensive understanding of the intended adoption of new modes. For example, suppose that a WSB or another innovative school travel mode is introduced. In that case, parental attitudes and satisfaction toward this new option may initially be superficial or not exist at all due to limited knowledge/experience. Moreover, outcome evaluations such as attitudes, satisfaction, and desire for the innovative school travel mode are underdeveloped, and they cannot be measured or included in existing theories. Nonetheless, parents’ and children’s intentions toward the new mode may depend on how they evaluate existing modes. In this line, a positive attitude toward current active travel modes may correlate with a favourable intention to try WSB, and desire to use a car for school trips may correlate negatively with it. This distinction is necessary as most of the theoretical models and empirical evidence available in the literature regarding the TPB, MGB and TCD are associated with behavioural intention towards the use of existing modes with which people have some level of familiarity (e.g., car, walk, public transport). These missing aspects can be explained, as De Vos et al. ^57^ indicate, by researchers’ preference for studying stated intention through stated choice experiments rather than considering it as a latent construct. Therefore, adaptations regarding the object (mode) to which each construct is measured need to be proposed if understanding intended mode choice adoption is the research aim.

The lack of integration of these theories has also been recognised in the literature, and the narrow scope criticised^57^. For instance, even though the MGB is proposed as an extension of the TPB, instead of proposing both direct and indirect effects (the latter mediated by desire), it removes the direct effects between attitude, PBC and subjective norms with behavioural intention. De Vos et al.^57^ after an extensive literature review on socio-psychological constructs on mode choice, recognised these limitations, proposing an integrated framework called the Travel Mode Choice Cycle (TMCC). The approach connects intention, mode choice, satisfaction, attitude, and desire, establishing a theoretical base for analysing a more comprehensive set of relationships between these constructs, including direct and indirect effects. Nonetheless, efforts of this kind are limited in the literature, and empirical evidence is scarce. Therefore, there is an opportunity to continue existing efforts by adding the necessary empirical evidence for assessing more comprehensive, integrated conceptual models.

## Method

### Study area

This study was conducted with parents of primary school-aged children residing in the Bradford district, UK. Bradford is located in West Yorkshire in northern England and spans approximately 370 square kilometres. It is the fifth largest metropolitan borough council in England, with a population of around 563,600^84^. The district is characterised by a highly diverse ethnic composition—with the Asian or Asian-British population rising from 26.8% in 2011 to 32.1% in 2021, and the White British population declining from 63.9% to 56.7% over the same period—and pronounced socio-economic inequalities. It is the 13th most deprived local authority in England and has a higher proportion of economically inactive residents (27.1%) compared with the national average (21.4%). Among inactive individuals aged 16–64, 21.9% are not in employment because they are responsible for looking after the family or home^85^. Together, these demographic and socio-economic factors shape complex school travel patterns within the local context. As evidenced in local “School Streets” initiatives, streets around schools are frequently congested, creating safety risks and limiting opportunities for children to walk^86^. In parallel, contemporary urban development in Bradford—including investment in walking and cycling infrastructure and programmes supporting sustainable travel to school—provides a favourable setting for structured walking interventions^87^.

While Bradford has distinct characteristics, including its ethnic diversity, higher-than-average deprivation levels, and local transport priorities, it also shares challenges common to many UK and international cities. These include congested streets around school areas, a higher share of car use for school journeys, and ongoing prioritisation toward active travel infrastructure. As such, Bradford offers a meaningful case through which to understand the design and implementation of WSB programmes in complex, high-need urban environments. Nonetheless, aspects such as local sociocultural norms, neighbourhood-level deprivation, and specific policy drivers (either at the school or council level) may limit direct transferability, and these contextual elements should be considered when applying our findings to other settings.

### Data collection

Ethical approval was granted by the University of Leeds Ethics Committee (reference number: BESS+ FREC 0682). The approval process involved submission of a detailed protocol outlining the study design, recruitment approach, data-collection procedures, data-management plan, participant consent form, and a fieldwork risk assessment. Approval required that the study avoid collecting personally identifiable information and not directly involve vulnerable groups. Consequently, children were not surveyed or asked to provide any information, and the survey did not ask for identifiable details such as the home address or child-level demographic information. Only postcode data were collected to calculate approximate home-to-school distance. Support was offered to those needing assistance with the survey, and participants could withdraw at any time. These safeguards ensured anonymity and compliance with ethical standards for minimal-risk research.

Parents of primary school-aged children were focused on in this study because the choice of school travel mode is largely determined by parental decisions at this stage^19^. Moreover, existing literature highlights that encouraging children to adopt green transport habits early in life maximises their social learning potential and fosters the long-term adoption of active travel in daily routines^88^. In England, primary school age children are aged from 4/5 years to 11.

During the pilot phase of the survey, the questionnaire link was shared with parents from schools in Bradford that were either part of the Clean Air Schools Programme (CASP), coordinated by the City of Bradford Metropolitan District Council (CBMDC), or engaged in other school-related projects run by Born in Bradford (BiB). Additional pilot-phase dissemination occurred through the research team’s professional networks. In the final phase, survey outreach was expanded to maximise parent engagement. The survey poster link was circulated via community organisations working closely with parents—specifically the BEAP Community Partnership (via its Facebook group) and the Nigerian Community Association in Bradford (NCAB) (via its WhatsApp group). Further dissemination was supported by a list of schools provided by CBMDC and BiB, as well as schools whose parent communities engaged through BEAP and NCAB.

The project team liaised with school authorities—specifically targeting schools where the WSB programme had not yet been implemented. In total, 21 schools voluntarily disseminated the first part of the questionnaire, which focused on children’s current school travel patterns and parents’ socio-demographic profile, through their newsletters and parent communication channels. Paper-based questionnaires were also made available upon request. The link to the second part of the questionnaire—addressing the WSB and parents’ psychological factors—was embedded at the end of the first-part of the questionnaire so that respondents could continue seamlessly. However, if any responses were incomplete or missing, the research team subsequently redistributed the second-part of the questionnaire directly to parents using the email addresses collected during the first part of the questionnaire. Eligible participants were exclusively parents residing in Bradford with at least one child attending primary school. Participating schools disseminated the survey link, an awareness-raising video (detailing the structured WSB programme), the consent form, and the research participant privacy notice through their parents’ communication channel.

In the survey, three units of observation were used: parent-level variables, child-level school travel-pattern variables, and household-level variables. As children were not involved in the survey, all information was reported by parents. The first part of the questionnaire, distributed by schools through their parents’ communication channels, collected information on the responding parent’s demographic profile (e.g., age, gender, education, employment status, ethnicity, and possession of a driving licence), household characteristics (e.g., car ownership, number of household members, number of children in the household, number of children studying same primary school, and access to subsidised public transport), and the child’s current school travel patterns. These travel-pattern items included: child’s usual mode of travel for the home-to-school and return journeys, modes used during adverse weather conditions (e.g., during heavy wind or rain), escorting arrangements, and school tour type. When responding to the school-travel-plan questions, parents were able to indicate whether caregivers, relatives, or neighbours were involved in accompanying the child to school as part of the escorting options. The second part of the questionnaire focused solely on parent-level information. This section asked parents to evaluate a series of statements designed to measure key psychosocial latent and observed constructs relevant to the study.

A total of 212 responses were received; however, 192 were deemed valid for analysis. Responses were excluded if the participant had not completed the entire questionnaire or had taken part in the survey after the WSB programme had been introduced in their child’s school. To maintain a control condition, only responses from parents who had no prior experience with the WSB were included. Upon completing the survey, all respondents received a small gift from the project team. Additionally, ten parents were selected to receive a £25 gift voucher through a raffle draw. The survey administration process was closely coordinated with school administration teams to ensure smooth implementation and avoid disruption to academic activities. Furthermore, although there is no universally agreed rule for determining sample size in SEM, it is generally accepted that a minimum of 100 cases is required, with sample sizes above 200 considered ideal to ensure more reliable model estimation^89^. A sample is typically deemed adequate when SEM significance tests and model fit indices can be interpreted reasonably^90^. Moreover, for this sample size of 192, the margin of error for the worst-case was ≈± 7.1 (at 95% confidence level and worst-case proportion *P* = 0.5), offering reasonable precision for population estimates^91^.

**Fig. 1:**
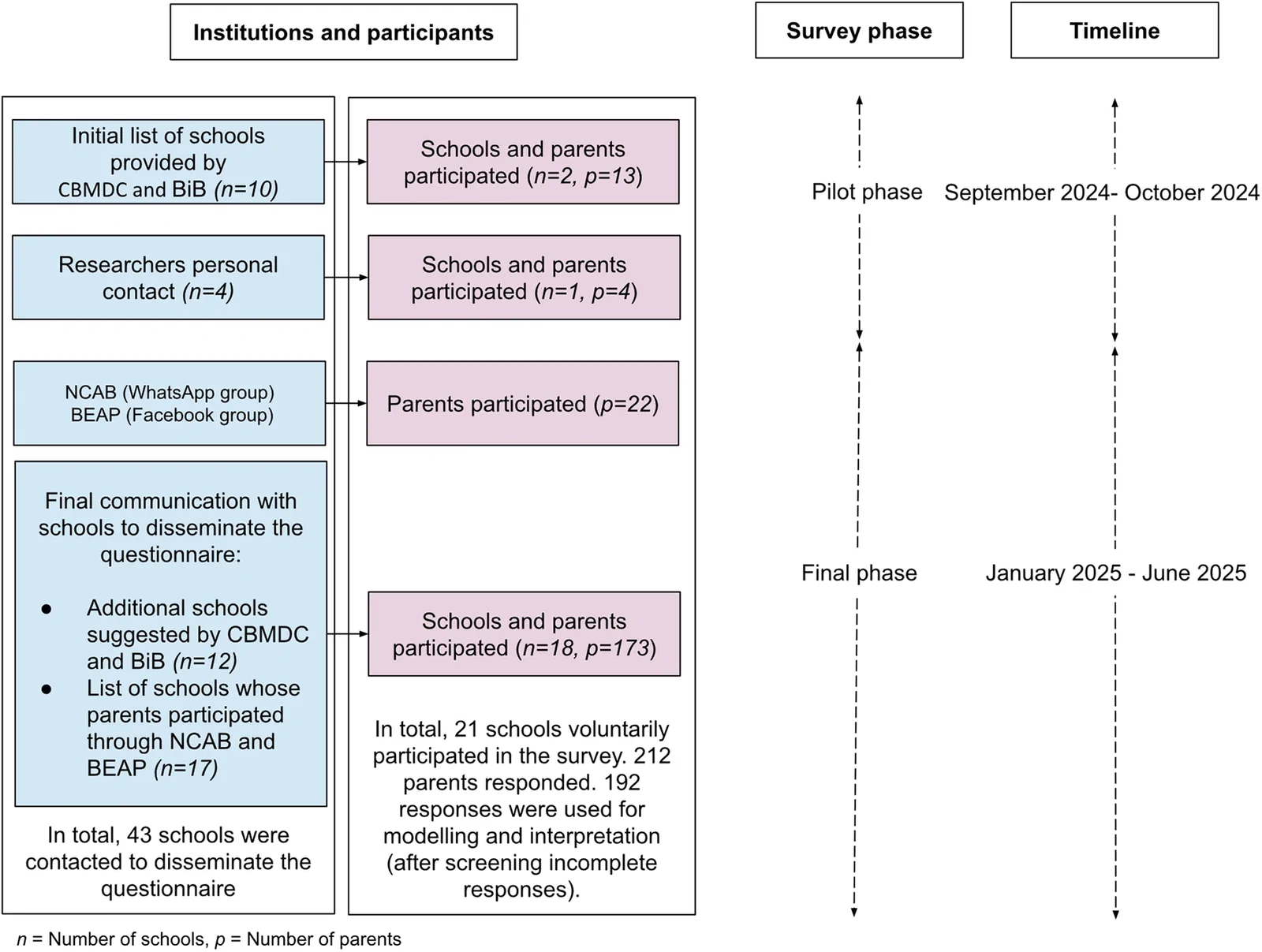
Flowchart summarising the recruitment process and data-collection procedure. This figure illustrates the survey process adopted in this study, including key activities such as school recruitment, participant enrolment, and the timelines of the survey phases. The left panel ("Institutions and participants'') summarises how schools were identified and contacted through multiple channels. The middle panel details participation outcomes across these recruitment streams. The right panel presents the survey timeline divided into two phases. Together, the figure provides a comprehensive overview of recruitment pathways, participation rates, and the temporal structure of the study.

Moreover, the survey phases, corresponding timelines, and the involvement of schools and parents at each stage are presented in Fig. 1.

### Sample characteristics

The sample data comprised both male (20%) and female (80%). The majority were aged between 35–44 years (61.8%), followed by those aged 25–34 (27.2%). In terms of educational attainment, over 34.4% of respondents had completed a bachelor’s degree, while 17.2% held a master’s, MPhil, or PhD qualification. A majority of respondents (40.1%) reported working full-time, while others indicated working part-time (25.4%), being unemployed and seeking work (9.6%), and not working (13.56%). Ethnically, 14.5% reported as White (including English, Irish, Roma, or other White), and 63% reported as Asian or Asian-British. Additionally, 71% of respondents reported possessing a driving licence. Regarding household composition, 72.1% of respondents reported having four or fewer members in their household, while 27.9% reported a household size of five or more. A substantial majority (76.4%) reported having more than one child under the age of 15 in the household, and 43.2% had more than one child attending the same school. 98.5% of the respondents had access to at least one car in the household. However, only 10.4% reported that someone in their household had free access to public transportation, either due to being a senior citizen or pension age, having a qualifying disability, having children aged below 5 years old, holding a bursary-related free bus pass, or having a family member employed by a public transport company. The details of sample characteristics can be found in Table 2.

**Table 2:** Sample characteristics

Variable	Total (*n* = 192)
**Respondent level variables (parent)**	Percentage (%)
**Gender**	
Male	20.11
Female	79.89
**Age (years)**	
<18	1.05
18–24	0.52
25–34	27.23
35–44	61.78
45–54	7.85
>55	1.57
**Education**	
Primary	4.17
GCSE	19.27
A-level	13.02
Bachelor (1st to 2nd year)	8.33
Bachelor (up to final year)	26.04
Masters/Mphill	15.63
PhD	1.56
Other	11.98
**Employment**	
Working full-time	40.11
Working part-time	25.42
Looking for work (unemployed)	9.60
Not working (not looking for work)	5.65
Not working due to health reasons	7.91
Other	7.91
**Ethnicity**	
White (English, Irish, Roma, other white)	14.52
Asian or Asian-British	63.44
Black, Black British, Caribbean or African	11.83
Mixed or multiple ethnic groups	6.45
Others	3.76
**Driving license**	
Yes	70.90
No	29.10
**Household-level variables**	
**Number of underage (<15) children in the household**	
1	23.56
2	45.03
3	19.90
>3	11.52
**Number of children studying in the same primary school**	
1	56.77
2	33.33
>=3	9.90
**Number of cars at the household**	
0	0.53
1	35.26
2	44.74
>2	19.47
**Household size**	
≤4	72.11
>4	27.89
**Free access to public transport service**	
Yes	10.4
No	89.6

In the questionnaire, parents were also asked to report on their child’s school travel characteristics (see Table 3 for details). As the survey was disseminated via specific schools and parents participated through their child’s school communication channels, the travel-related responses corresponded to the school journey of that particular child. No travel-related information was collected for any other children in the household who were enrolled in different schools. For parents participating via community organisations, they were required to mention their child’s primary school at the beginning of the survey, and all subsequent questions pertained to that child. Among the 192 valid responses from parents, 42.2% of children frequently walked to school, 53.1% were driven by car, and 4.2% used public transport (bus). The remaining respondents used other available modes, such as cycling, ride-sharing, or ride-hailing. For the return journey from school to home, walking increased slightly to 44.3%, while 49.5% returned by car. Between 2002 and 2023, national travel survey statistics indicated that the main school travel modes for children aged 5–10 were walking (47–53%) and car travel (39–47%)^92^. Hence, the collected data reflect the average school travel characteristics currently prevailing in the UK. Furthermore, mode choice was reported during adverse weather conditions, with 28.7% choosing to walk and 66.2% using a car. Among respondents, 67.7% reported making a round trip from home to school and back home, while 21.9% accompanied their child on the way to work. When asked who typically escorted the child to school, 44.3% reported it was solely the mother, 19.3% the father, and 29.2% reported that both parents were involved in the school drop-off. The remaining cases involved caregivers, siblings, friends from the same neighbourhood, or other relatives living nearby. While car use and walking were the dominant modes of school travel, the data revealed significant diversity in terms of who accompanied the child, how the trip was made, and how the school journey was integrated into the parents’ daily travel routines.

**Table 3:** Children’s school travel statistics (responded by parents)

Variable	Total (*n* = 192)
	Percentage (%)
**Current school travel mode choice (usual weather condition)**	
(Home to School)	
Walking	42.19
Car	53.13
Bus	4.17
Other	0.52
**Current school travel mode choice (usual weather condition)**	
(School to home)	
Walking	44.27
Car	49.48
Bus	4.17
Other	2.08
**Current school travel mode choice (adverse weather condition)**	
Walking	28.65
Car	66.15
Bus	4.69
Other	0.52
**Preferred school travel mode choice**	
Walking	45.83
Car	48.44
Bus	4.17
Other	1.56
**Escorting type**	
Child’s father	19.27
Child’s mother	44.27
With both father and mother	29.17
Child’s caregiver	0.52
Other siblings studying in the same primary school	0.52
Child’s friend living in the same neighbourhood	0.52
Relatives living/sharing the same house	0.52
Relatives living in the same neighbourhood	1.56
Other	3.65
**School travel routine**	
I/My partner/Someone from home accompanies my child at school and comes back home	67.71
I/My partner/Someone from home accompanies my child at school on the way to work	21.88
I/My partner/Someone from home accompanies my child on the way to grocery or personal activities	2.08
My child goes with other siblings studying in the same primary school	2.60
My child goes with other “children/friends” living in the same neighbourhood and studying in the same primary school	2.08
My child is going to school alone	1.56
Other	2.08

### Research hypotheses and indicators

**Fig. 2:**
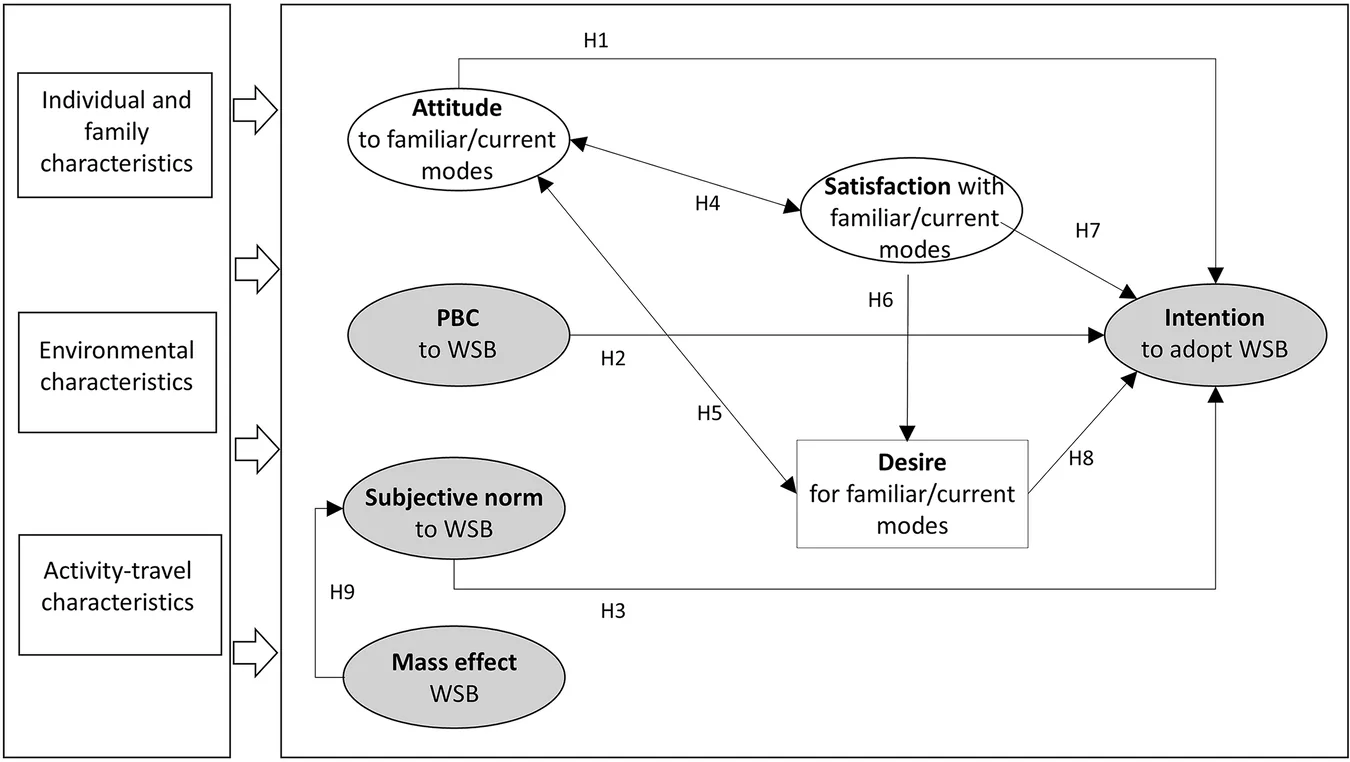
The proposed intended mode choice adoption (IMCA) conceptual model for WSB. This figure presents the conceptual framework underpinning the study, illustrating the relationships between psychological constructs and the intention to adopt WSB. Individual and family characteristics, environmental characteristics, and activity-travel characteristics are shown as exogenous factors influencing the main constructs within the model. Psychological variables shown in grey are related to WSB, and those shown in white are related to the existing mode. Ovals represent latent variables (unobserved constructs measured through indicators), while rectangles denote observed variables. Arrows indicate hypothesised causal paths (H1-H9), with arrow direction showing the proposed influence between constructs. Exogenous variables are positioned on the left, and endogenous variables are organised progressively towards the right, culminating in behavioural intention.

Based on insights from previous AST research, identified gaps in the literature, and the specific characteristics of the WSB, this study proposes a conceptual framework that examines parents’ stated intention to adopt WSB. The corresponding research hypotheses are embedded within this overall framework and are illustrated in Fig. 2. The starting point for its development was the integration of constructs and relationships from the TPB, MGB, TCD and related studies. In this way, the conceptual framework highlights the role of *satisfaction*, *desire*, *attitude*, *perceived behavioural control (PBC)*, *subjective norms (SN)* and *mass effect* in explaining *WSB intention*.

The conceptual framework also incorporates the specificities needed to integrate those constructs into WSB adoption. In particular, specifying attitudes, satisfaction, and desire as direct determinants of intention to use the WSB may not be feasible in the context of this study. The WSB is not yet fully operational in the case study, as it is currently operating at a limited scale in a few primary schools in Bradford, funded by the CBMDC as a pilot project. Having that in mind, attitudes, satisfaction, and the desire to WSB would be hypothetical and unrealistic, because intention toward WSB (an unfamiliar mode for most parents) has not yet been developed. Thus, attitudes, satisfaction, and desire were incorporated, but toward modes that are familiar to parents (e.g., car, walk, public transport). In the case of attitude, it was oriented toward active travel and assessed using a combination of behavioural beliefs and outcome evaluation^93^. Satisfaction was assessed based on parents’ experiences with their current school travel plan. This approach was taken for two main reasons: (1) to examine whether satisfaction with other alternative school travel modes influences parents’ intention to switch to the WSB, and (2) to understand how satisfaction with the current travel plan reflects parents’ attitudes toward existing active travel modes. Satisfaction with the current school travel plan was measured using six statements. To understand parents’ desired mode of school travel, they were first asked about their current mode of travel, followed by their most preferred mode under different circumstances, such as usual conditions and adverse weather conditions. As a result, in this study, the desire for car use for school travel was treated as the observed variable, reflecting the frequency with which parents prefer the car over other available alternatives for school trips.

On the other hand, PBC and SN were oriented to capture parents’ pre-conceived social and psychological beliefs about WSB. This is possible because PBC captures parents’ perceptions of ease or difficulty in allowing their children to participate in WSB, focusing on initial identification of potential barriers and enablers. PBC, which refers to an individual’s belief in their ability to perform a behaviour and their personal sense of control over it^50^, was assessed among parents, focusing on their perception of whether their child is capable of walking with other children and whether they, as parents, have the flexibility to adapt to the WSB schedule. Similarly, in this study, SN refers to parents’ perceptions of the value they place on other’s behaviour, opinions, communication, or experiences if the WSB were to be introduced. To capture this dimension, parents were asked three questions about whether they believed that people close to them would want them to use the WSB for their child. An important characteristic of the adopted conceptual model is that, despite considering the relationships stated in the MGB, PBC and SN were set not to affect desire. The basis for this distinction relates to the object each construct focuses on: desire was directed towards a familiar/current mode, while PBC and SN were directed to the emerging mode (WSB). Therefore, it would not be theoretically correct to maintain these connections in the model, which is why only the direct effects between them and behavioural intention were included in the proposed conceptual model. Finally, the proposed conceptual model also introduced a latent variable affecting SN called “mass effect”. The mass effect was introduced as an alternative and innovative way of operationalising descriptive norms within the conceptual framework. It reflected the influence of observing or hearing about other parents’ actions and experiences with the WSB and was posited to impact SN by shaping what is perceived as acceptable or encouraged behaviour^56^.

Therefore, along with information on parents’ socio-economic status and their child’s school travel plan, parents responded to several statements designed to capture key psychological latent and observed variables. All statements were adapted from previous relevant studies^93–96^. Response options were measured using 5-point Likert scales with different wordings, including: (A) *Very unlikely* to *Very likely*, (B) *Not sure at all* to *Very certain*, (C) *Not at all* to *Very strongly*, and (D) *Strongly disagree* to *Strongly agree*. A table mapping constructs, survey items, response options and descriptive statistics is shown below in Table 4.

**Table 4:** Description and descriptive statistics of the indicators included in the survey

Variables and Indicators	Response	Mean	SD
***Intention*** *to use WSB*			
INT1: Will you allow your child to use walking school bus regularly?	A	2.96	1.39
INT2: How probable is it that you’ll choose the Walking School Bus for your child’s next school trip?	B	3.38	1.17
INT3: Do you believe the use of a walking school bus can positively impact your child’s overall school experience (e.g., social learning, discipline, independence)?	C	3.55	1.08
INT4: Do you agree it’s a good idea to include the Walking School Bus in your child’s school travel plan, considering your comfort and convenience?	D	3.78	0.96
***Attitude*** *towards active mode*			
ATT1: I prefer walking, cycling or public transport as I am concerned about my child’s health and well-being	D	3.43	1.20
ATT2: I want my child to become familiar with an environmentally friendly mode (walking/cycling/public transport)	D	3.97	0.96
ATT3: Even if I have access to a car, opting for walking or cycling during school trips provides valuable physical exercise for both me and my child	D	4.11	1.00
ATT4: I consider the connectivity between home and school through walking, cycling, and public transport to be of greater importance than good facilities for cars (on-street/off-street free parking)	D	3.49	1.05
ATT5: When my child is travelling with me, I prefer to take public transport rather than drive whenever possible	D	2.46	1.17
*Perceived behavioural control* ***(PBC)***			
PBC1: How easy or hard would it be for your child to use the Walking School Bus?	E	3.31	1.08
PBC2: How easy or hard would it be for you to get used to the Walking School Bus?	E	3.35	1.07
*Subjective norm* ***(SN)***			
SN1: If the Walking School Bus is introduced, do you think it is important to get opinions from neighbours while deciding to use it (as the service will be operating in your neighbourhood)?	C	3.01	1.23
SN2: When considering the Walking School Bus, do you believe the opinions of relatives and friends are important to you?	C	2.53	1.37
SN3: Do you think your partner would support your decision if you choose the Walking School Bus for your child?	C	3.71	1.12
*Positive* ***mass effects***			
PM1: Do you think the opinions of the parents already using the Walking School Bus are important to you?	C	3.41	1.27
PM2: Would their good or bad experiences impact your decision to choose it?	C	3.14	1.27
PM3: When deciding on the Walking School Bus, do you care about other parents’ decisions who haven’t used the service?	C	2.35	1.25
***Satisfaction*** *with current travel plan*			
ST1: I am happy with how my child is currently going to school	D	4.14	1.14
ST2: I am choosing the best travel mode	D	4.15	1.05
ST3: I am choosing the safest home-to-school route	D	4.43	0.92
ST4: I do not want to change anything in the current travel plan	D	3.66	1.37
ST5: Current school travel plan makes me feel like a good parent	D	4.19	1.07
ST6: My child is very happy with the current travel plan	D	4.22	1.10
***Desire*** *for a car for school travel*			
Counting the number of times respondents selected the car as their preferred mode in these scenarios (at present/most preferred/during adverse weather conditions), reflecting their overall inclination toward using a car for school travel.	*N*/*A*	*N*/*A*	*N*/*A*

### Structural equation modelling

The research hypotheses were evaluated using structural equation modelling (SEM), which is a flexible technique used in different disciplines to analyse complex relationships among observed and latent (unobserved) variables. It combines elements of factor analysis and multiple regression, allowing researchers to assess direct and indirect relationships between variables within a conceptual framework. Usually, SEM has two components: (1) a measurement model, and (2) a structural model. This study involved both since our list of variables includes both observed and latent variables. A measurement model (using confirmatory factor analysis) specifies how observed variables (either exogenous or endogenous) are related to their respective latent variable (*ξ*, *η*). Equation (1) has been used for the measurement model of exogenous latent variables (*X*):

1 $$X={\Lambda }_{x}\xi +\delta$$

Here, *X* = Vector of observed exogenous indicators, *Λ*_*x*_ = Factor loading matrix for exogenous variables, *ξ* = Vector of exogenous latent variables, *δ* = Measurement error for exogenous indicators. Equation (2) has been used for the measurement model for endogenous latent variables (*Y*):

2 $$Y={\Lambda }_{y}\eta +\epsilon$$

Here, *Y* = Vector of observed endogenous indicators, *Λ*_*y*_ = Factor loading matrix for latent variables, *η* = Vector of endogenous latent variables, *ϵ* = Measurement error for endogenous variables.

Moreover, through a Multiple Indicators Multiple Cause (MIMIC) model, the resulting factors are able to be evaluated in terms of their influence on the dependent variable (Equation 3). Thus, the MIMIC model takes into account the influence of observed variables on latent variable.

3 $$\eta =\Gamma X+\zeta$$

Where, *η* = Vector of endogenous latent variables, *X* = Vector of observed exogenous variables (direct causes of *η*), *Γ* = Matrix of regression coefficients (how strongly each *X* affects each *η*), *ζ* = Structural disturbance term.

All the SEM models were estimated using AMOS version 30. The assessment of the models goodness-of-fit was done using traditional indicators in SEM, such as, Root Mean Square Error of Approximation (RMSEA), Comparative Fit Index (CFI), Tucker–Lewis Index (TLI) and the variance explained.

## Results

### Measurement model

To assess the proposed conceptual model, firstly, Cronbach’s alpha coefficients were calculated prior to model estimation to assess internal consistency. The calculated alpha values reported in Table 5 show that all exceeded or were near the recommended threshold of 0.7^97^, indicating acceptable reliability. Then, a preliminary CFA was conducted, including all the indicators associated with each latent variable, and the standardised factor loadings were assessed. The thresholds used in this study follow the guidelines proposed by Hair et al.^97^, with the following model fit criteria: (1) Standardised Factor Loading (SFL) of 0.5 or higher, (2) Average variance extracted (AVE) of 0.5 or greater, and (3) Construct reliability (CR) of 0.7 or greater. AVE is a measure of convergent validity representing the average proportion of variance explained by a latent construct across its indicators. An AVE of 0.5 or greater indicates adequate convergent validity. CR is a measure of internal consistency and reliability of the indicators representing a latent construct. A CR of 0.7 or higher is typically considered acceptable. Accordingly, to enhance reliability and reduce measurement error, one indicator from attitude towards active travel variables (ATT5: When my child is travelling with me, I prefer to take public transport rather than drive whenever possible), and one indicator from subjective norm variable (SN3: Do you think your partner would support your decision if you choose the Walking School Bus for your child?) were removed. Regarding the latent variable satisfaction, due to strong correlation among its indicators, the measurement model collapsed, leading to the use of a single indicator instead of six to represent the construct. As a result, the satisfaction variable was included in the final model as an observed variable to overcome the identification issue.

**Table 5:** Standardised parameters of measurement model with convergent validity. Cronbach’s alpha in squared parenthesis

Variable	Indicator	SFL^*a*^	SE^*b*^	CR^*c*^	AVE^*d*^
Intention to use WSB [0.87]	INT1	0.99	0.09	0.88	0.64
	INT2	1.00	0.07		
	INT3	0.86	0.07		
	INT4	0.83	0.06		
Attitude towards active mode [0.82]	ATT1	0.79	0.08	0.81	0.54
	ATT2	0.88	0.06		
	ATT3	0.77	0.06		
	ATT4	0.63	0.07		
Perceived behavioural control (PBC) [0.87]	PBC1	0.88	0.07	0.88	0.78
	PBC2	1.00	0.06		
Subjective norm (SN) [0.74]	SN1	1.12	0.13	0.75	0.61
	SN2	0.89	0.13		
Mass effects [0.65]	ME1	0.97	0.14	0.57	0.34
	ME2	0.73	0.13		
	ME3	0.37	0.11		

*χ*^2^: 172.13, df = 80 (*p* < 0.001) Comparative Fit Index (CFI): 0.928, Tucker–Lewis Index (TLI): 0.906, Loglikelihood (*LL*_0_): −3866.587, Loglikelihood (*LL*_1_): −3780.523, Akaike Information Criterion (AIC): 7813.175, Bayesian Information Criterion (BIC): 7943.475, RMSEA: 0.07.

^a^*SFL* standardised factor loading.

^b^*SE* standard error.

^c^*CR* composite reliability.

^d^*AVE* average variance extracted.

A summary of standardised parameters of the measurement model, along with the evidence of convergent validity, is reported in Table 5. The model demonstrated acceptable goodness-of-fit indices. Specifically, the CFI and TLI exceeded the recommended threshold of 0.90, and the RMSEA was 0.07, which is between 0.03 and 0.08 at 95% level of confidence^97^. Although the Chi-square statistic (*χ*^2^ = 172.13 and *df* = 80) was significant, the ratio of Chi-square to degrees of freedom (*χ*^2^/*df* = 2.15) was below the recommended threshold of 5, indicating a reasonable model fit^98^.

As shown in Table 5, all the standardised factor loadings of indicators were statistically significant at the 95% confidence level. CR values ranged from 0.75 to 0.88, exceeding the minimum threshold of 0.70, with the exception of the mass effect variable, which had a CR of 0.57. Similarly, the AVE values for all latent variables ranged from 0.54 to 0.78, surpassing the recommended cut-off of 0.50, again with the exception of mass effect, which had an AVE of 0.34.

It is important to note that one indicator from the mass effect variable (ME3: When deciding on the WSB, do you care about other parents’ decisions who haven’t used the service?) was retained in the final measurement model, despite its SFL and AVE slightly below 0.50 and its CR falling below the threshold of 0.70. This decision was based on the consistency index for mass effect (0.65), which is marginally below the acceptable threshold, and the AVE values for all variable pairs exceeded the squared correlations between the variables, thereby providing evidence of discriminant validity (see Table 5 and Table 6). These results suggest that the latent variables exhibit adequate internal consistency and that the measurement model demonstrates good convergent and discriminant validity. Overall, the hypothesised measurement model is both reliable and appropriate for testing the structural relationships among the latent variables. Furthermore, the overview of indicators data for latent variable construction has been reported in Table 4.

**Table 6:** Square of the correlation between latent variables

Latent constructs	Attitude	SN	PBC	Mass	Intention
Attitude	1.000				
SN	0.011	1.000			
PBC	0.031	0.028	1.000		
Mass	0.025	0.227	0.164	1.000	
Intention	0.121	0.047	0.545	0.190	1.000

### Structural model

**Fig. 3:**
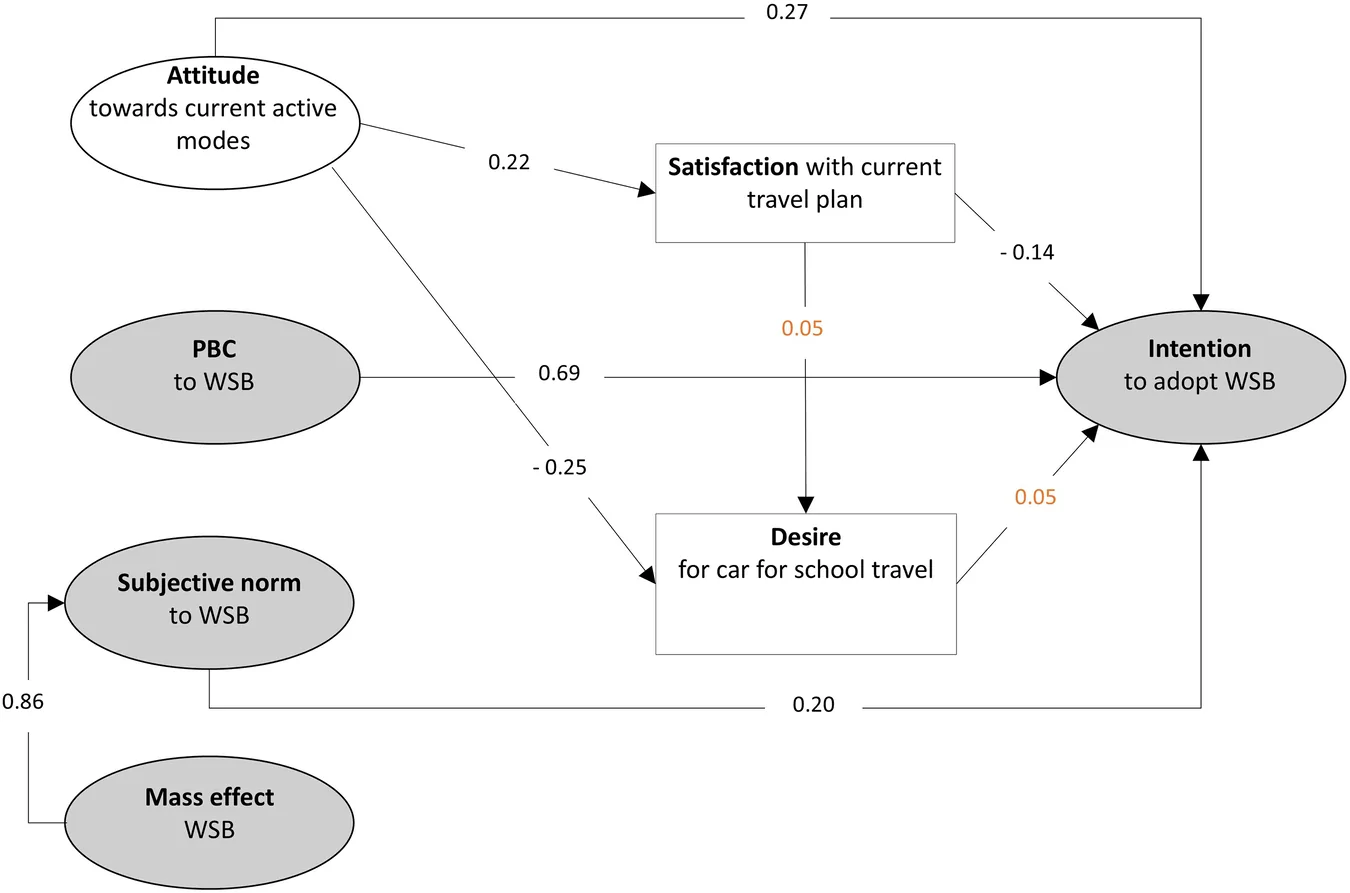
Standardised path coefficients of SEM model. This figure presents the estimated SEM model linking psychological constructs to the intention to adopt WSB, with standardised path coefficients displayed along each relationship. Path significance is indicated by colour coding of the coefficients: black values represent relationships significant at the 95% confidence level, blue values indicate significance at the 90% level, and yellow values denote statistically insignificant paths.

After having a good fit of the CFA model, the validity of the structural model and its corresponding hypothesised theoretical relationship was also tested. Using the same indices used for CFA, we found that the structural model had a reasonable fit (*χ*^2^: 242.337, df = 110 (*p* < 0.001), CFI: 0.900, TLI: 0.877, RMSEA: 0.07). The standardised path coefficient and their statistical significance have been reported in Fig. 3. Moreover, the details of model fit and model estimates of the IMCA SEM model are reported in Table 7. The IMCA SEM model explained 58% of the variance in behavioural intention to switch to WSB. It is worth noting that, due to the cross-sectional nature of the data, the current study examined only the unidirectional relationships within the proposed IMCA model, rather than attempting to assess bidirectional effects.

**Table 7:** Standardised regression weights (S.Est.), standard error (S.E.), critical ratio (C.R.) and model fit statistics of SEM and SEM-MIMIC model

	SEM			SEM-MIMIC		
Path	S.Est.	S.E.	C.R.	S.Est.	S.E.	C.R.
Attitude towards current active modes → Intention	0.273	0.083	3.829	0.271	0.082	3.843
PBC to WSB → Intention	0.685	0.091	7.953	0.677	0.091	7.901
Subjective norm to WSB → Intention	0.203	0.121	2.423	0.215	0.120	2.698
Satisfaction with current travel plan → Intention	- 0.135	0.058	-2.277	-0.132	0.058	-2.230
Desire for a car for school travel → Intention	0.048	0.045	0.824	0.042	0.044	0.719
Satisfaction with current travel plan → Desire for a car for school travel	0.054	0.094	0.747	0.084	0.088	1.220
Attitude towards current active modes → Satisfaction with current travel plan	0.222	0.092	2.853	0.225	0.091	2.923
Attitude towards current active modes → Desire for car for school travel	-0.251	0.123	-3.137	-0.214	0.114	-2.838
Mass effect WSB → Subjective norm to WSB	0.862	0.163	3.798	0.843	0.145	3.720
Ethnicity (British) → Satisfaction with current travel plan				0.121	0.190	1.725
Car available at the household and driving license → Attitude towards current active modes				-0.140	0.133	-1.820
Postgrad qualification → PBC to WSB				0.128	0.172	1.708
Female → Subjective norm to WSB				-0.212	0.176	-1.816
Mother accompanies children for school travel → Mass effect WSB				-0.212	0.55	-2.646
Multiple underage children (>1) at the household → Desire for a car for school travel				0.221	0.176	3.321
Home-to-school distance (>1 mile) → Desire for car for school travel				0.216	0.195	3.238
Younger-aged parents (<25 years old) → Desire for car for school travel				-0.126	0.650	-1.895
**Goodness-of-fit indices**						
*χ*^2^	242.337			478.093		
*df*	110			266		
*CFI*	0.900			0.853		
*TLI*	0.877			0.834		
*AIC*	328.337			596.093		
*BIC*	468.409			788.283		
*RMSEA*	0.07			0.065		

An analysis of standardised estimates confirmed that the hypothesised relationships within the proposed IMCA conceptual model were mostly statistically significant, except for the paths between satisfaction with the current school travel plan and desire for school travel, and between desire for car use for school travel and intention to adopt WSB. Consistent with TPB, in terms of effects on intention to switch to WSB, the estimates of the standardised coefficients showed that attitude towards current active mode (*β* = 0.27), PBC (*β* = 0.69) and SN (*β* = 0.20) towards WSB were all positive and significant at the 95% confidence level.

Moreover, complying with the proposition of the cognitive model, attitude towards the current active mode was positively and significantly associated with satisfaction with the current school travel plan (*β* = 0.22). Also, following the extension proposed by the MGB model, attitude towards current active mode was negatively associated with the desire for a car for school travel (*β* = −0.25). An extension introduced in the proposed IMCA conceptual model, beyond the existing state-of-the-art frameworks, was the inclusion of parents’ mass effect in explaining the variance of SN. This effect was found to be positive and statistically significant (*β* = 0.86), suggesting that the collective influence or perceived behaviour of other parents plays a meaningful role in shaping individual parents’ perceptions of social expectations regarding WSB adoption.

Interestingly, when comparing the strength of the individual path coefficients, PBC towards WSB emerged as the most influential factor in explaining the variance in parents’ intention to use WSB, followed by attitude, SN, and satisfaction with the current school travel plan. The findings indicated that the inclusion of mediating psychological variables such as satisfaction enhances the model’s ability to capture the complexity of switching behaviour. Furthermore, both factors directly related to WSB—such as PBC and SN—and those associated with current travel modes—such as attitude and satisfaction—influenced a parent’s intention to transition from their current mode to WSB.

### SEM-MIMIC model

Although the SEM model can estimate the direct, indirect, and total effects, it cannot distinguish between subgroups within the sample unless it is partitioned. Such variation in the assessment of latent variables is made possible by a MIMIC model. MIMIC model can test many variable groupings simultaneously and eliminates the need to divide the sample during the modelling. Therefore, in this study, SEM-MIMIC models were estimated to detect heterogeneity in the measurement of both latent and observed variables defined in the IMCA framework, arising from individual and family characteristics, environmental factors, and activity-related variables.

**Fig. 4:**
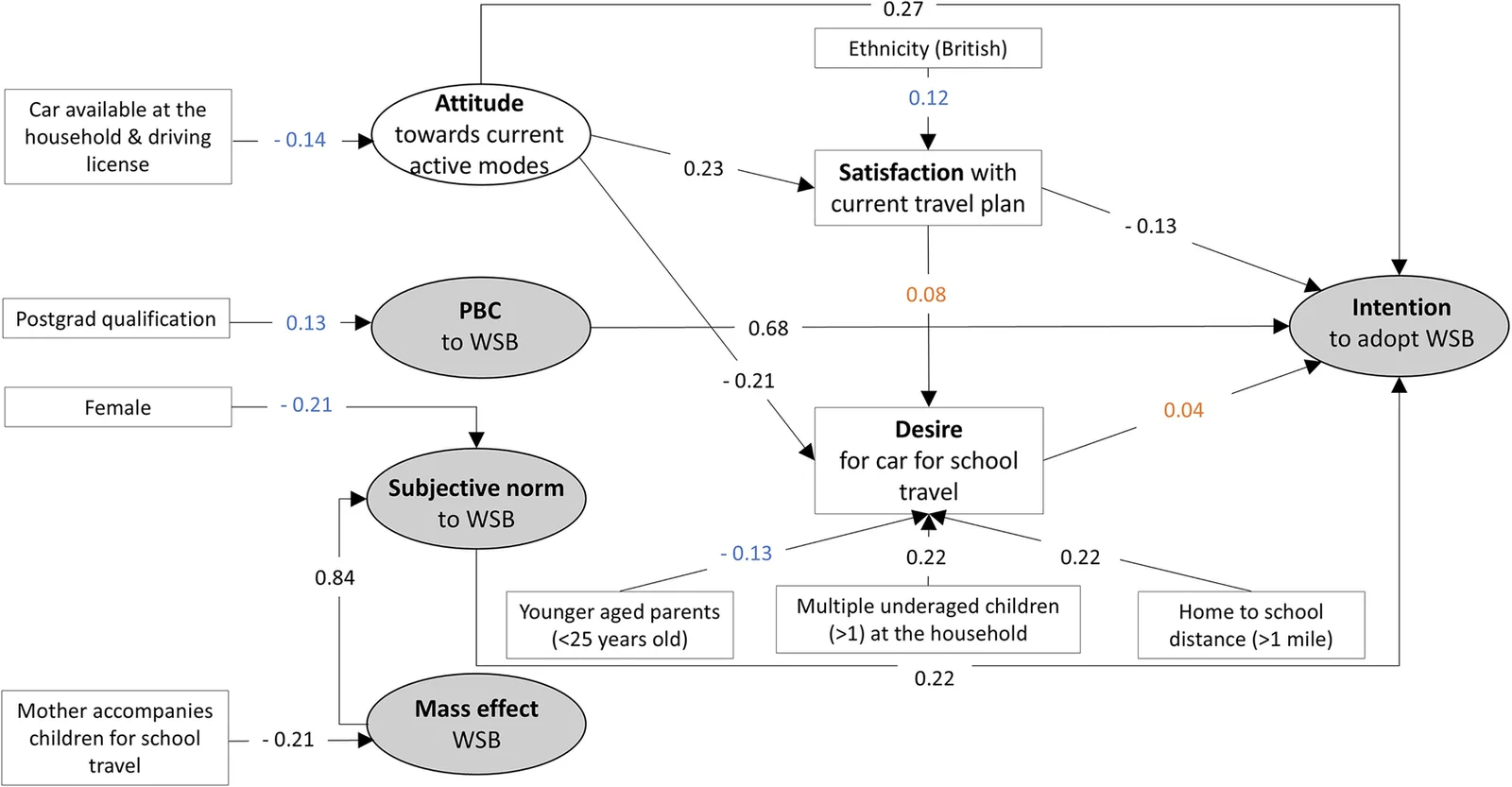
Standardised path coefficient of SEM-MIMIC model. This figure presents the SEM-MIMIC model incorporating socio-demographic, household, and travel-related observed variables alongside latent psychological constructs to explain intention to adopt WSB. Path significance is indicated by colour coding: black coefficients are significant at the 95% confidence level, blue coefficients at the 90% level, and yellow/orange coefficients denote statistically insignificant relationships.

Variables included in this study comprised of individual characteristics such as parent’s age, gender, ethnicity, employment status, driving license, and education level; family characteristics such as car ownership, the number of under-aged children (<15 years old) at the household, and the number of children attending the same primary school; and trip attributes such as the distance from home to school and escorting type. These socio-demographic variables were specified as direct causes of the latent and observed variables and included in the model as binary variables, consistent with previous studies exploring the impact of socio-demographic factors on latent attitudes and perceptions. Figure 4 presents the standardised coefficients of socio-demographic variables and their effects on the variables used in hypothesis testing. Table 7 includes the model fit and estimates of the IMCA SEM-MIMIC model. The model fit indicated a marginal improvement in RMSEA; however, the SEM-MIMIC specification was retained due to its theoretical relevance in capturing heterogeneity in mode-switching intentions across socio-demographic groups. Results highlighted that, similar to the SEM model, most standardised path coefficients between socio-demographic and latent/observed variables were statistically significant.

Among all variables, having more than one under-aged children at the household was strongly associated with a higher desire to use a car for school travel (*β* = 0.22), compared with households that have only one child. The effect of having multiple children attending the same primary school was also examined. However, due to the high correlation between the number of children in the household and whether those children attended the same school, only one of these variables was retained in the final model. Notably, when multiple children in a household attended the same school, parents exhibited a lower desire to use a car for school travel. These results reflect the dual scenario of parental time constraints. For example, having more than one child in a household can create complex travel patterns, such as different school locations and schedules depending on each child’s age, resulting in multiple drop-offs or pick-ups and additional household obligations. Such time constraints may be reduced when children attend the same school, as similar schedules and locations simplify travel demands. These findings were further supported when considering the distance from home to school (>1 km). Longer home-school distances were positively and significantly associated with parents’ desire for car use for school travel (*β* = 0.22), suggesting that longer distances likely increase parents’ need for time management. Interestingly, parents’ age was also found to influence the desire for car use. Younger-aged parents (< 25 years old) showed a lower propensity to desire a car for school travel compared to older adults. This could be related to their income levels, car ownership, or different attitudes towards sustainable mobility.

Beyond car desire for school travel, the influence of gender was also assessed in relation to parents’ SN toward WSB use (*β* = −0.21). The model found that female respondents exhibited lower SN in favour of WSB, potentially due to greater protective instincts and reliance on personal judgement over social influence. In line with this result, when mothers escorted their children for school travel, they had relatively lower positive mass effects compared to other groups (e.g., escorted by father, siblings, caregiver, or relatives/neighbour) (*β* = −0.21), likely due to mothers’ protective norms and heightened safety anxiety. Education was found to have a significant positive effect on PBC toward WSB (*β* = 0.13). This finding suggests that individuals with more advanced education may feel more confident in engaging with the structured WSB system, potentially due to their financial stability, differences in access to information, familiarity with emerging services, managerial skills or cognitive approaches to evaluating new mobility options.

When examining attitudes toward active travel, households with car availability and parents holding a valid driving licence were found to exhibit relatively higher negative attitudes toward active modes of travel (*β* = −0.14). This finding is intuitive because for people with access to a car and a driving licence, car use becomes the most convenient and time-efficient option for daily routines. As a result, they may perceive less need or benefit in adopting active modes such as walking or cycling.

Also, the effect of ethnicity indicated that for white British parents, satisfaction with their current school travel plan was higher as compared to the other ethnic group (*β* = 0.12). This implies a relatively lower propensity of this group to adopt WSB, as satisfaction with the current travel plan was negatively associated with intention to adopt WSB. Differences in satisfaction levels between White British parents and parents from other ethnic groups may stem from a range of multidimensional factors—such as income variation, accessibility to preferred travel options, living conditions, and perceptions of safety and security.

Overall, parents’ intention to switch to a new mode such as the WSB was ultimately shaped by a combination of latent and observed psychological variables related to both emerging and conventional modes, which were themselves influenced by socio-demographic factors and practical constraints such as distance and household obligations.

## Discussion

WSB, as a sustainable school travel mode, has the potential to overcome the limitations of conventional active travel options (e.g., walking or cycling) and reduce the negative externalities associated with private car use for school commutes. However, understanding parents’ intention to switch to such an emerging mode is essential in determining how quickly the programme will be accepted and adopted, as intention has been found to directly influence behaviour^28^. This study examined the interplay of factors influencing parents’ intention to switch to the WSB by proposing an IMCA conceptual model that not only incorporates WSB-specific variables but also considers other available travel alternatives. Such investigation is necessary before the development of a robust model to predict WSB demand and inform targeted interventions to promote its use by addressing parental psychological constructs, demographic factors, and built environment variables.

Findings from this study highlighted that understanding the demand for the WSB is not straightforward, as intention—a key determinant of demand—was shaped by a multilayered interaction of psychological, demographic, and environmental factors that influence each other synergistically. The results supported the validity of the proposed IMCA framework and theoretical assumptions derived from the TPB, MGB, and TCD. Among the TPB constructs, PBC had the strongest influence on intention to switch to WSB, thereby supporting Hypothesis H2. This aligns with previous research on AST (e.g., walking or cycling), which also identified PBC as a key determinant of intention to use active modes for school travel^40^. However, the WSB model presents several practical and contextual constraints that may limit its feasibility or scalability, which in turn may reduce parents’ and children’s confidence in using it, increase perceived barriers, and weaken their sense of control. Prior studies have noted challenges such as coordinating parental or children’s schedules with fixed WSB meeting times, and the limited suitability of WSB for children with certain special educational or physical needs^9,14,99^. Another potential barrier is the infrequent operation of many WSB programmes, often due to a limited number of participating children, shortages of volunteers, insufficient school involvement, and inadequate funding^3,6,8,100^. This inconsistency may reduce its suitability for families who require reliable, daily school-travel arrangements. Additional external barriers may include adverse weather conditions, safety concerns related to existing infrastructure (e.g., cross-walks, walkways), traffic around the school/neighbourhood^99,100^, and WSB route-specific characteristics such as total route distance, spatial distribution of routes, or the distance from home to the nearest WSB stop^2,3^. Therefore, to support potential WSB adoption, it is essential to address the practical constraints that would shape parents’ and children’s confidence in their ability to use this emerging mode—and their perceived sense of control over doing so—in future WSB promotion guidelines.

Moreover, the results showed that attitude towards other available active travel modes and SN were both significant predictors of intention, supporting Hypotheses H1 and H3. This finding contrasts with several studies on AST, which reported the insignificance of either attitude or SN in predicting intention—often explained by the limited volitional autonomy of young children and parental safety concerns^40,44,49,50,67^. However, for the WSB, both attitudes toward active travel and SN toward WSB emerged as significant determinants of intention to switch to this mode. It could be because of the supervised travel provision in WSB under adult guidance and adherence to safety protocols. Yang et al.^17^ also suggested that interventions aimed at improving attitudes toward AST were a prerequisite for the successful implementation of WSB initiatives. Unlike traditional forms of active travel, social approval and influence from others may play a more prominent role in shaping parental intention to adopt an emerging and relatively unfamiliar mode such as the WSB. The importance of SN is further supported in this study by the positive and significant influence of the mass effect on SN, consistent with Hypothesis H9. Two mechanisms may help explain the relationship between SN and mass effects, as well as the relationship between SN and the intention to adopt WSB. First, when considering an emerging mode designed to address concerns associated with conventional school-travel options (e.g., safety), parents may seek wider social acceptance to validate their decision. Prior research similarly suggests that to increase the uptake of active travel modes, social support should extend beyond parents alone^44^. Second, because the WSB operates as a participatory and frequently volunteer-driven initiative, a sense of collective ownership is crucial for its effective functioning and long-term sustainability. Insufficient social support or volunteer engagement can jeopardise programme continuity^99,100^, especially given the dynamic nature of school communities, where WSB routes require periodic adjustment as new families join and others leave.

In line with assumptions from the MGB, satisfaction was found to significantly mediate the relationship between attitude and intention, supporting Hypothesis H4 and H7. The direct paths from attitude to intention and from attitude to satisfaction to intention were both significant, suggesting that satisfaction partially mediated the attitude-intention relationship. However, given the opposing direction of the mediated path, this reflected a suppression effect—whereby parents with a positive attitude toward active travel were more likely to be satisfied with their current travel arrangements, but this satisfaction in turn dampens their intention to adopt the new form of walking as part of WSB. Despite this, attitude continued to exert a direct positive effect on intention, reinforcing the notion that rational evaluations (e.g., health, environmental concern) may override emotional attachment when considering alternatives. Furthermore, satisfaction with other existing travel alternatives, not just the WSB itself, is important for understanding parents’ intention to adopt an emerging mode. Parents’ satisfaction with conventional alternatives may create habitual inertia, leading to a stronger intention to continue using the conventional mode rather than switching to the new one. As emphasised by Wang et al.^18^, the long-term success of WSB schemes depended not only on service quality but also on children’s enjoyment and parental satisfaction. Furthermore, while attitude toward active modes was found to negatively affect the desire for car-based school travel, the path from desire to intention was statistically insignificant. This suggested that desire for car use may not mediate the relationship between attitude and intention, rejecting Hypothesis H8. In other words, even if some parents express a low desire for car use, this does not necessarily translate into a behavioural intention to adopt the WSB in the school travel context. Instead, it may simply reflect a preference for continuing with conventional active modes such as walking or cycling.

It is important to note that, although the current study did not examine the bidirectional relationship proposed by the IMCA, the findings provided intuitive insights underscoring its significance. For instance, the desire for car use in school travel did not significantly influence the intention to adopt the WSB; it was shaped by attitudes toward current active modes. If, cyclically, car-use desire subsequently influences attitudes toward active travel, this could reduce the overall propensity to adopt the WSB. A similar pattern could be observable in the attitude-satisfaction pathway, suggesting that improving attitudes toward active travel may simultaneously enhance satisfaction and decrease reliance on the car, thereby increasing the likelihood of considering WSB participation. Conversely, the suppressing effect of satisfaction with the current school-travel plan on intention to adopt the WSB highlighted the importance of fostering satisfaction with the WSB service itself—an area warranting further investigation. Without satisfaction in the new service, even parents with positive attitudes toward active travel may remain committed to their existing travel arrangements. Thus, while improving attitude toward active current active mode is essential for promoting WSB adoption, it is equally important to mitigate factors that diminish intention. These findings suggest a dual-strategy approach: (1) Target psychological constructs (attitude, perceived behavioural control, subjective norms) that directly influence intention, (2) Address competing factors—such as satisfaction with existing modes and desire for car use—that indirectly undermine these constructs.

The results further revealed the influence of various socio-demographic factors on the psychological constructs shaping parents’ intention. These included both family characteristics (e.g., number of underage children, parental access to a car and having a driving license) and individual factors (e.g., parental education, gender, ethnicity). For instance, respondents with access to a car and a driving license tend to demean parents’ attitudes toward active travel, which in return likely to reduce the propensity to adopt WSB and increase their desire for car use for school travel, as they may find it convenient to drop off their children due to other time commitments such as household obligations or work pressure^65^. However, a study by Seraj et al.^47^ found no significant association between parental attitudes toward active modes and car ownership. This is likely because, in the context of Southern California, car ownership was nearly universal, with almost every household owning at least one vehicle. In contrast, the present study considered both household car access and possession of a driving license, revealing a sizeable proportion of respondents (approximately 30%) which reflected the influence of access to a car on parents' attitudes.

Moreover, results from this study also suggested that the presence of more than one under-aged child in the household, parents aged greater than 24 years and a greater home-to-school distance (>1 mile) encouraged further desire for a car, reflecting the complex travel demand pattern. While other studies on AST have highlighted household characteristics—such as family obligations, number of children, household size, and the mother’s occupation—as significant determinants of parental decisions to drive or walk to school^101^, this study's findings revealed how those factors contributed to desire for a car for school travel. Nevertheless, household constraints often discourage walking and, in turn, contribute to a stronger preference for car use, driven by convenience and a diminished attitude toward active travel^61,65^. Therefore, when designing WSB systems, it is important to account for variability in household demographics and to provide services that address these constraints in order to stimulate greater demand for WSB. Furthermore, the WSB model—by offering designated stops closer to residential areas or integrating park-and-ride/drop-off options with WSB stoppage facilities within walking distance of schools—may help alleviate issues related to distance as well as parents' time constraints and safety concerns^102,103^.

Moreover, results from this study revealed that among the individual factors, higher parental education was associated with greater PBC toward using the WSB. The effect of education on PBC to WSB aligns with the findings of Amireault et al.^104^, who empirically demonstrated that income is a key moderator of the PBC-behaviour relationship. Given the widely acknowledged correlation between higher education and higher income, it is reasonable to infer that those who tend to have greater financial stability may also be more engaged in, and invested in, their children’s educational and developmental activities. This, in turn, may increase their willingness to consider alternative school-travel arrangements that they perceive as beneficial. Parents with higher levels of education may also have greater exposure to institutional communication channels and be more familiar with school-based organisational processes, which could enhance their sense of control when evaluating an emerging programme such as the WSB. Additionally, higher educational attainment is often associated with employment opportunities that develop managerial and organisational skills. Such skills may influence how parents interpret information, assess risks, and navigate school-related initiatives, contributing to differences in PBC between parents with higher and lower levels of education. Given that PBC is a modifiable construct, understanding how education shapes PBC is crucial for designing strategies to support mode shift. Beyond education, prior studies have shown that PBC can be strengthened through information provision, guided experience, and supportive communication—for example, practical demonstrations, clear explanations of WSB procedures, and opportunities for parents to trial the service may enhance perceived control across all educational groups^100^. WSB guidelines should therefore recognise the diverse educational backgrounds of parents and their varying levels of PBC, ensuring that promotional strategies are accessible and equitable to support broader uptake across different demographic groups.

Another key finding from this study was the significant influence of gender on SN toward WSB and on the escorting effect for positive mass. This aligns with the findings of Dill et al.^51^, who observed that women expressed lower support for walking and cycling initiatives. Similar patterns were identified in this study, with women reporting lower SN toward WSB. In addition, when mothers accompanied their child, a lower positive mass effect was observed. One possible interpretation is that mothers may exhibit reduced confidence in the reliability of close social ties when making decisions about their child’s travel, potentially due to heightened safety concerns or more protective orientations compared with fathers. This highlighted the importance of addressing these concerns, given that maternal attitudes are often key determinants of AST, as demonstrated by Corral-Abós et al.^46^.

While many studies have demonstrated a significant association between ethnicity and AST^73,105^, the results of this study reveal notable differences in satisfaction levels between White British individuals and other ethnic groups. Satisfaction was found to be a key determinant of the intention to adopt WSB and was negatively associated with adoption intention. Consequently, White British participants—who reported higher satisfaction with their current travel arrangements—were less likely to switch to WSB. This finding suggests that non-White British individuals may represent potential adopters of WSB. This aligns with previous research on AST indicating that families from non-White ethnic backgrounds are more likely to engage in AST than their White counterparts^70,106^. However, the literature also reports mixed findings. Studies considering factors such as levels of acculturation, living conditions (e.g., distance from school), and household income have shown lower levels of AST among some minority ethnic groups^105,107^. Therefore, the role of ethnicity in the adoption of WSB warrants more critical and contextualised investigation across different ethnic groups. The likelihood of WSB adoption among ethnic minority populations may depend on intersecting factors such as residential location, socio-economic status, and degree of acculturation. These factors may reduce participation in active travel, including WSB. To break the potential vicious cycle whereby improvements in socio-economic status and residential mobility contribute to a shift away from AST, policy measures should focus on increasing satisfaction with active travel modes among different ethnic groups. Enhancing satisfaction is essential for promoting WSB adoption, and similar principles may apply to the broader uptake of other active travel modes.

Overall, this study highlights the importance of factors related to both current and emerging travel modes in understanding parental intentions to switch to WSB. It also provides a foundational understanding of how individual characteristics, household constraints, and physical and social environmental factors shape and moderate the psychological determinants underlying behavioural intention to adopt WSB.

## Conclusion

The proposed conceptual framework successfully highlighted the significance of the hypothesised relationships in a unified structure for understanding parents’ intention to switch to WSB. While the study revealed important associations among psychological, demographic, and contextual factors, its findings can be further refined in future research—particularly by applying the framework in settings where a WSB is already operational. This would provide a more realistic context for validating the model and capturing parental perceptions based on direct experience. For example, if data had been available from users of WSB in a case where WSB is operational, it would have been straightforward to investigate, following the proposed conceptual framework, how users’ satisfaction with the current WSB service is affecting the desire for other alternatives like a car for school travel or further affecting other psychological constructs such as attitude, PBC and SN. Though this study, in its current form, did not explore such potential, it did confirm the validity of factors in the conceptual framework—not only related to the primary mode of concern but also to complementary modes such as satisfaction with the current travel plan or desire for a car for school travel—so as to appropriately capture the mediating effect. If the conceptual framework had been based solely on one theoretical framework to capture the mediating effect, it might have underestimated the direct effects of attitude, PBC, and SN in predicting intention.

The bidirectional relationships proposed in the IMCA framework were only partially tested due to the cross-sectional nature of the data. A follow-up study involving a pilot implementation of the WSB, followed by redistribution of the questionnaire, would allow for the collection of longitudinal data. This, in turn, would enable a more comprehensive investigation of the full IMCA framework, including the assessment of potential bidirectional relationships. Moreover, the influences of desire and satisfaction with the current school travel plan were investigated as observed variables. In future research, additional indicators could be examined to include them as latent variables in the IMCA framework to provide further insights into other relevant aspects.

The explanatory variables used to explain the latent constructs in this study were primarily based on parents’ individual and household characteristics. Because the survey data were collected anonymously, only the postcode and parents’ email addresses were recorded to track responses across the entire process of the survey. Including a child’s demographic information, combined with postcode data, could have made respondents personally identifiable. For this reason, child-related demographic variables (e.g., age, gender) were omitted. This limitation restricted the ability to examine how children’s age-specific needs and levels of independence may influence parental intentions to adopt WSB. For example, parents may perceive lower safety risks or have greater confidence in older children compared with younger ones, which could directly affect their willingness to adopt WSB. Other relevant contextual factors, such as parents’ perceptions of neighbourhood safety and safety within the school catchment area, were not included in the analysis. These perceptions are likely to vary according to children’s demographic characteristics and parents’ socio-economic conditions. In addition, the Asian-British parents were over-represented in the sample, which may affect the generalisability of the study findings. As such, caution is required when interpreting the results for the broader population and across different socio-demographic and contextual settings. Although this study accounted for those who escort children during school travel, it did not investigate in detail the decision-making dynamics related to childcare and upbringing within households. In particular, potential inequalities in caregiving responsibilities—such as gendered divisions of care, additional caregiving duties at home, or variations linked to household economic status—were not examined. These factors may deter parental participation in participatory travel arrangements such as WSB. Inclusion of child-specific characteristics, together with gender, caregiving burden, neighbourhood safety, and social vulnerability, while also aiming for more balanced and representative samples in future WSB research, would enable a more nuanced understanding of the determinants of WSB adoption and support the development of more inclusive and targeted interventions.

The mass effect and its influence were measured when the WSB was not operational. The effects of mass communication among parents and stakeholders are dynamic and tend to evolve over time. Future research should investigate how mass communication relates to the effectiveness of WSB operations in meeting school travel needs, and how perceptions of service quality influence WSB uptake during its initial implementation and after it has been in operation for some time. The positive and negative effects of mass communication ultimately shape social norms, which are direct determinants of the intention to switch to WSB. This highlights the need for dynamic models in future research to account for such cyclical effects when examining parents’ school travel mode choice behaviour.

Furthermore, the scope of this study was limited to examining intention to switch to WSB. Future research should investigate the direct links between intention, habit and behaviour, as these are essential for understanding the full complexities of WSB uptake. This may involve conducting a stated preference experiment in the current context—an approach we plan to pursue in our next phase of research. Alternatively, if data on both WSB users and non-users are available, the proposed conceptual framework could be applied to examine the relationships between intention, behaviour, and habit using either advanced econometric modelling or data-driven machine learning approaches. Finally, although some recent studies on AST have emerged from countries in Asia and Latin America, the Global South remains strongly under-represented compared to the Global North. Future research should therefore prioritise context-sensitive approaches and expand empirical evidence in diverse geographic settings to improve the generalisability of findings. This is relevant as reported effectiveness of interventions such as WSB programmes in the Global North may reflect institutional capacity, governance structures, and resource availability that differ significantly from the Global South.

## Data Availability

Due to ethical approval conditions and the need to protect participant confidentiality, the raw data underlying this study are not publicly available and are restricted to the core research team. However, aggregated data and fitted model outputs can be shared with researchers upon reasonable request to the corresponding author, subject to institutional and ethical approval.
